# *N*-*tert*-Butyl-2-{2-[2-(4-chloro­phen­yl)-4-hy­droxy-1-(5-methyl­isoxazol-3-yl)-5-oxo-2,5-di­hydro-1*H*-pyrrol-3-yl]-*N*-(4-meth­oxy­phen­yl)acetamido}-2-(4-meth­oxy­phen­yl)acetamide methanol monosolvate: single-crystal X-ray diffraction study and Hirshfeld surface analysis

**DOI:** 10.1107/S2056989021011312

**Published:** 2021-11-02

**Authors:** Mariia O. Shyshkina, Yana I. Sakhno, Oleksandr V. Radchenko, Svitlana V. Shishkina, Sergey M. Desenko, Valentyn A. Chebanov

**Affiliations:** a Division of Chemistry of Functional Materials, State Scientific Institution "Institute for Single Crystals" of the National Academy of Sciences of Ukraine, 60 Nauky Ave., Kharkiv 61072, Ukraine; b Faculty of Chemistry, V.N. Karazin Kharkiv National University, 4 Svobody Sq., Kharkiv 61077, Ukraine

**Keywords:** Ugi reaction, multicomponent reaction, mol­ecular structure, crystal structure, Hirshfeld surface analysis

## Abstract

The title compound, C_36_H_37_ClN_4_O_7_·CH_3_OH, which crystallizes as a methanol solvate, may possess biological activity, which is inherent for a natural peptide or protein. In the crystal, mol­ecules of the title compound form hydrogen-bonded tetra­mers with the solvate mol­ecules acting as bridges as a result of the O—H⋯O and N—H⋯O inter­molecular hydrogen bonds. Hirshfeld surface analysis was used to study the different types of inter­molecular inter­actions whose contributions are: H⋯H = 53.8%, O⋯H/H⋯O = 19.0%, C⋯H/H⋯C = 14.8%, Cl⋯H/H⋯Cl = 5.3%, N⋯H/H⋯N = 3.2%.

## Abstract 2

The mol­ecular and crystal structures of the title compound, C_37_H_41_ClN_4_O_8_, which has potential biological activity have been studied. This compound exists in the crystal phase as a methanol solvate.

## Chemical context

The combined application of Doebner and Ugi-type multicomponent reactions, with the participation of the azoloazine type of carb­oxy­lic acid as an acid component in the Ugi reaction to increase the mol­ecular diversity of the target heterocyclic compounds, was reported in our previous publication (Murlykina *et al.*, 2019 ▸). 
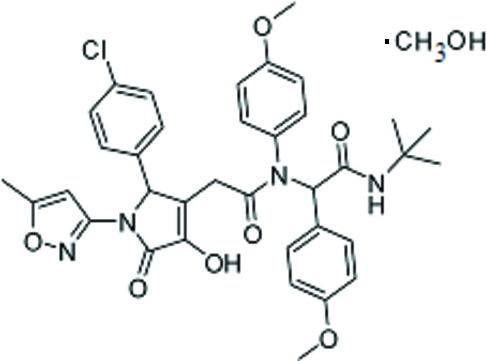


In the current work, the final product was synthesized *via* the four-component Ugi reaction of 2-[2-(4-chloro­phen­yl)-4-hy­droxy-1-(5-methyl­isoxazol-3-yl)-5-oxo-2,5-di­hydro-1*H*-pyr­rol-3-yl]acetic acid, 4-meth­oxy­aniline, 4-meth­oxy­benz­alde­hyde and *tert*-butyl­isocyanide. The target product contains a heterocyclic core bound to peptidomimetics, compounds that mimic a natural peptide or protein and which may have high biological activity. The pyrrolone fragment is also a privileged motif because of its biological activities, namely anti­bacterial (Murlykina *et al.*, 2013 ▸), anti­viral (Murlykina *et al.*, 2015 ▸; Rashid *et al.*, 2012 ▸; Pace *et al.*, 2008 ▸), anti­tumor (Mori *et al.*, 2013 ▸; Koz’minykh *et al.*, 2002 ▸) and anti­microbial (Khalaf *et al.*, 2004 ▸; Gein *et al.*, 2006 ▸).

## Structural commentary

**Figure 1:**
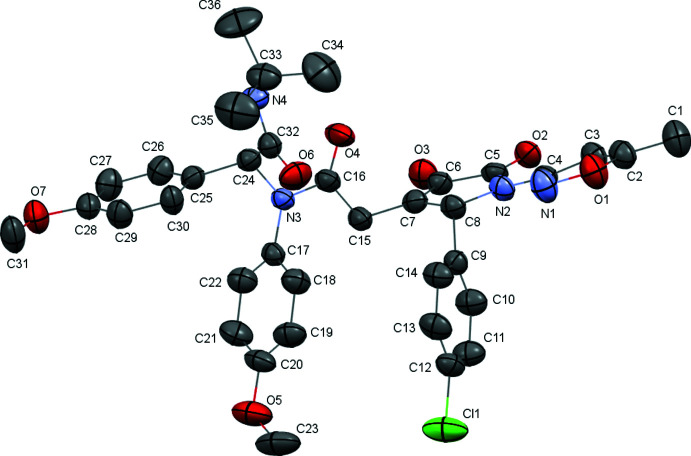
Mol­ecular structure of the title compound (solvent molecule and hydrogen atoms are omitted for clarity). Displacement ellipsoids are shown at the 50% probability level.

The title compound crystallizes as a methanol solvate (Fig. 1 ▸). The methanol mol­ecule is disordered over two positions (*A* and *B*) with the populations of *A*:*B* in a 0.303 (10):0.697 (10) ratio. All atoms of the partially saturated five-membered heterocycle are in the same plane with an accuracy of 0.008 Å. The N2—C4 bond length of 1.380 (3) Å and the C8—N2—C4—N1 torsion angle of 2.2 (4)° indicate conjugation between the π-systems of the partially saturated and oxazole cycles. The *para*-chloro­phenyl substituent is located in the pseudo-equatorial position and is turned in relation to the C7—C8 endocyclic bond [C6—C7—C8—C9 = −120.9 (3)° and C7—C8—C9—C10 = 60.9 (3)°]. The C16(=O4)—N3 carb­amide group is located in the *-ac* position in relation to the C6—C7 endocyclic bond [C6—C7—C15—C16 = −107.2 (3)°], and the C16=O4 carbonyl group is slightly non-coplanar to the C7—C15 bond [C7—C15—C16—O4 = 22.5 (4)°]. The *para*-meth­oxy­phenyl substituent at the nitro­gen atom is turned almost orthogonally to the plane of the carbamide group [C16—N3—C17—C22 = −99.5 (3)°]. The *para*-meth­oxy­phenyl substituent at the carbon atom is located in a position inter­mediate between *sp* and *−sc* and is also rotated almost orthogonally to the plane of the carbamide group [the C17—N3—C24—C25 and N3—C24—C25—C26 torsion angles are −33.4 (3) and −83.4 (3)°, respectively]. In both *para*-meth­oxy­phenyl substituents, the meth­oxy group is coplanar with the plane of the aromatic ring [the C19—C20—O5—C23 and C29—C28—O7—C31 torsion angles are −3.3 (5) and 3.6 (4)°, respectively] despite the steric repulsion between the methyl group and the aromatic ring atoms (the shortened contacts are: C23⋯H19 = 2.52, H23*B*⋯C19 = 2.73, H23*A*⋯C19 = 2.76 and H29⋯C31 = 2.50, H31*C*⋯C29 = 2.77 and H31*B*⋯C29 = 2.70 Å as compared with the C⋯H van der Waals radii sum of 2.87 Å). The substituent at the C24 atom is located in the *−sc* position relative to the N3—C16 bond [C32—C24—N3—C16 = −78.1 (3)°] and the C32—O6 carbonyl group is slightly non-coplanar to the N3—C24 bond [O6—C32—C24—N3 = −27.8 (3)°]. The *tert*-butyl substituent is located in anti­perplanar position to the C32—C24 bond [C33—N4—C32—C24 = 172.9 (2)°].

## Supra­molecular features

**Figure 2:**
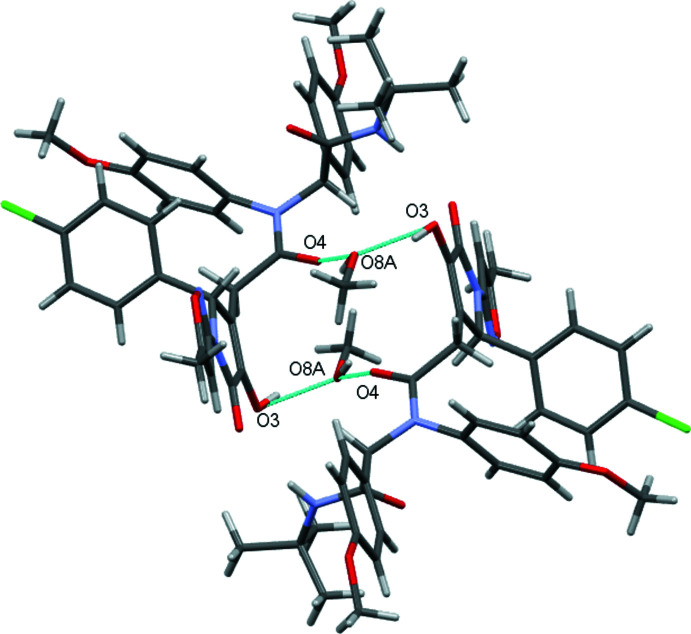
Tetra­mer of hydrogen-bonded title mol­ecules linked through methanol solvent mol­ecules.

In the crystal, the mol­ecules of the title compound are linked by bridging methanol mol­ecules due to the formation of the O3—H8*A*⋯O8*A*, O3—H8*B*⋯O8*B*, O8*A*—H8*A*⋯O4 and O8*B*–H8*B*⋯O4 inter­molecular hydrogen bonds (Table 1 ▸). Additionally, two main mol­ecules are bound by N4—H4⋯O2 hydrogen bonds (Table 1 ▸) within this dimer. As a result, a hydrogen-bonded tetra­mer may be recognized as a structural motif of the crystal packing (Fig. 2 ▸).

## Hirshfeld surface analysis

**Figure 3:**
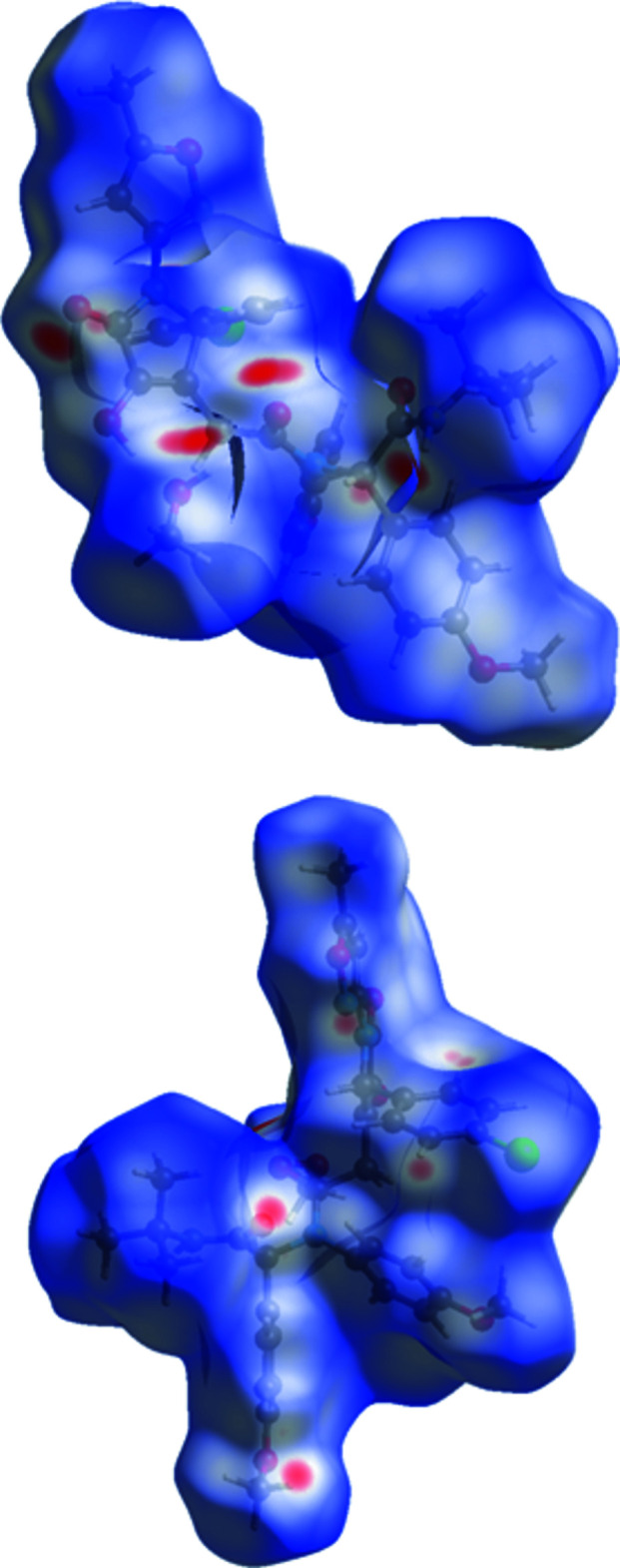
Two views of the Hirshfeld surface of the title mol­ecule mapped over *d_norm_* in the range −0.295 to 1.590 a.u.

Different types of intra- and inter­molecular inter­actions in a crystal structure can be identified and visualized with Hirshfeld surface analysis (Turner *et al.*, 2017 ▸). The mol­ecular Hirshfeld surface of the major compound was generated using a high surface resolution with three-dimensional *d_norm_* surfaces. The areas that are coloured red on the *d_norm_* surfaces correspond to contacts that are shorter than the van der Waals radii sum of the closest atoms (Fig. 3 ▸). These red spots indicate atoms participating in hydrogen bonding or short contacts. The brightest red spots are observed at the hydroxyl groups of both the main and the methanol mol­ecules, indicating a strong O—H⋯O inter­molecular hydrogen bond. In addition, bright-red spots are observed at the carboxyl group and at the hydrogen atom of the amino group, indicating short contacts. It should be mentioned that smaller red areas are found at the nitro­gen atom of the partially saturated five-membered heterocycle, at the C31H_3_ methyl group and at the C32=O6 carboxyl group, indicating short contacts.

**Figure 4:**
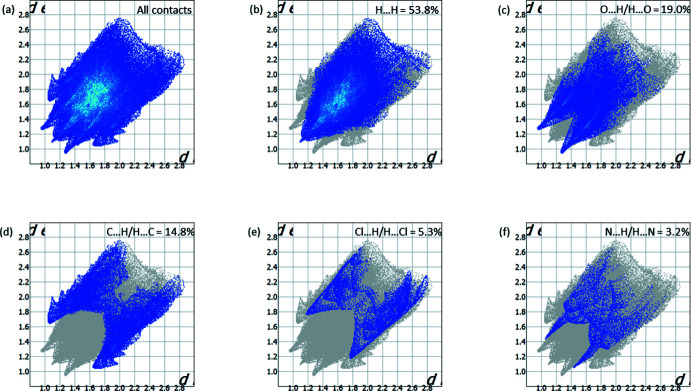
Two-dimensional fingerprint plot for the title compound showing (*a*) all inter­actions, and delineated into (*b*) H⋯H, (*c*) O⋯H/H⋯O, (*d*) C⋯H/H⋯C, (*e*) Cl⋯H/H⋯Cl. (*f*) N⋯H/H⋯N contacts.

In the two-dimensional fingerprint plots the pair of sharp spikes indicate strong hydrogen bonds and short contacts in the crystal structure (Fig. 4 ▸ *a*). The highest contribution is from H⋯H contacts (53.8%), while these made by the O⋯H/H⋯O (19.0%) and C⋯H/H⋯C (14.8%) inter­actions are similar (Fig. 4 ▸ *c*, 4*d*). The contributions of Cl⋯H/H⋯Cl (5.3%) and N⋯H/H⋯N (3.2%) inter­actions (Fig. 4 ▸ *e*, 4*f*) are very small.

## Database survey

A search of the Cambridge Structural Database (CSD Version 5.42, update of November 2020; Groom *et al.*, 2016 ▸) for the 3-hy­droxy-1,5-di­hydro-pyrrol-2-one fragment revealed 79 hits. Only 27 of these hits contain a fragment with the same structure as that of the title compound [refcodes: BOQXEN (del Corte *et al.*, 2019 ▸), CIKPAQ (Sarkar *et al.*, 2018 ▸), EVIYUD (Aliev *et al.*, 2003*b* ▸), GEJZAY (Mashevskaya *et al.*, 2011 ▸), GIMGEQ (Sarkar *et al.*, 2013 ▸), GITCAQ, GITDEV (Saha *et al.*, 2017 ▸), IRUBUS (Aliev *et al.*, 2003*a* ▸), LIFBEJ, LIFBOT (Sun *et al.*, 2011 ▸), NUXPIG (Wiedemann *et al.*, 2009 ▸), PADHUA (Zonouz *et al.*, 2015 ▸), PASTOT (Nicolaou *et al.*, 2005 ▸), QIPNAH (Bhajammanavar *et al.*, 2019 ▸), ROHNAG (Hosseinzadeh *et al.*, 2019 ▸), TOMPER (Sakhno *et al.*, 2008 ▸), UJEXOY (Ahankar *et al.*, 2016 ▸), VILQEP (Gein *et al.*, 2018 ▸), VIPNAJ (Mylari *et al.*, 1991 ▸), VIQDOP (Guseinov *et al.*, 2006 ▸), VOWGAP (Kaza­kov *et al.*, 1990 ▸), WAPMIM (Dubovtsev *et al.*, 2016 ▸), XINHIL (Aliev *et al.*, 2001 ▸), XOKRAT, XOKRAT01 (Ramazani *et al.*, 2019 ▸), YAJMOM (Wei *et al.*, 2004 ▸), YIYFAP (Denislamova *et al.*, 2014 ▸)]. All these structures and the title compound have the same electron density distribution within the 3-hydroxy-1,5-dihydro-pyrrol-2-one fragment.

## Synthesis and crystallization

**Figure 5:**
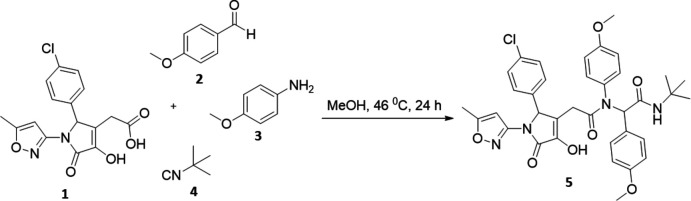
Reaction scheme.

4-Meth­oxy­aniline (**3**) (0.5 mmol) and 4-meth­oxy­benzaldehyde (**2**) (0.5 mmol) were dissolved in methanol (4 mL) and stirred for 0.5 h. Then, 2-[2-(4-chloro­phen­yl)-4-hy­droxy-1-(5-methyl­isoxazol-3-yl)-5-oxo-2,5-di­hydro-1*H*-pyrrol-3-yl]acetic acid (**1**) (0.5 mmol) and *tert*-butyl­isocyanide (**4**) (0.5 mmol) were added consistently and the reaction mixture was stirred for 24 h at 319 K. The mixture was allowed to stand overnight. The crystal precipitate was filtered off and dried. The reaction scheme is shown in Fig. 5 ▸.

## Refinement

Crystal data, data collection and structure refinement details are summarized in Table 2 ▸. All hydrogen atoms were located in difference-Fourier maps. They were included in calculated positions and treated as riding with C—H = 0.96 Å and O—H = 0.82 Å, *U*_iso_(H) = 1.5*U*_eq_(C,O) for methyl and hydroxyl groups and with Car—H = 0.93 Å, C*sp* ^3^—H = 0.97 Å, N—H = 0.89 Å *U*_iso_(H) = 1.2*U*_eq_(C,N) for all other hydrogen atoms. The solvent molecule is disordered over two positions (*A* and *B*) in a 0.303 (10):0.697 (10) occupancy ratio. C*sp* ^3^—O bonds were refined with fixed length of 1.413 Å.

## Supplementary Material

Crystal structure: contains datablock(s) I. DOI: 10.1107/S2056989021011312/zq2268sup1.cif

Structure factors: contains datablock(s) I. DOI: 10.1107/S2056989021011312/zq2268Isup2.hkl

Supporting information file. DOI: 10.1107/S2056989021011312/zq2268Isup3.cml

CCDC reference: 2118094

Additional supporting information:  crystallographic information; 3D view; checkCIF report

## Figures and Tables

**Table 1:** Hydrogen-bond geometry (Å, °)

*D*—H⋯*A*	*D*—H	H⋯*A*	*D*⋯*A*	*D*—H⋯*A*
O3—H3⋯O8*A*	0.75 (4)	1.88 (5)	2.61 (3)	162 (4)
O3—H3⋯O8*B*	0.75 (4)	1.93 (4)	2.680 (10)	175 (4)
N4—H4⋯O2^i^	0.87 (3)	2.33 (3)	3.193 (3)	170 (3)
O8*A*—H8*A*⋯O4^i^	0.82	1.88	2.60 (3)	145
O8*B*—H8*B*⋯O4^i^	0.82	2.50	2.843 (8)	107

Symmetry code: (i) -x+1, -y+2, -z+1.

**Table 2:** Experimental details

Crystal data	
Chemical formula	C_36_H_37_ClN_4_O_7_·CH_4_O
*M*_r_	705.19
Crystal system, space group	Triclinic, *P*\overline{1}
Temperature (K)	293
*a*, *b*, *c* (Å)	10.3211 (7), 13.9316 (11), 15.0269 (10)
α, β, γ (°)	107.179 (6), 100.769 (6), 109.649 (7)
*V* (Å^3^)	1843.2 (2)
*Z*	2
Radiation type	Mo *K*α
μ (mm^−1^)	0.16
Crystal size (mm)	0.12 × 0.08 × 0.04

Data collection	
Diffractometer	Rigaku Xcalibur, Sapphire3
Absorption correction	Multi-scan (*CrysAlis PRO*; Rigaku OD, 2018 ▸)
*T*_min_, *T*_max_	0.718, 1.000
No. of measured, independent and observed [*I* > 2σ(*I*)] reflections	12120, 6484, 3993
*R*_int_	0.062
(sin θ/λ)_max_ (Å^−1^)	0.595

Refinement	
*R*[*F* ^2^ > 2σ(*F* ^2^)], *wR*(*F* ^2^), *S*	0.063, 0.180, 0.98
No. of reflections	6484
No. of parameters	488
No. of restraints	14
H-atom treatment	H atoms treated by a mixture of independent and constrained refinement
Δρ_max_, Δρ_min_ (e Å^−3^)	0.37, −0.37

Computer programs: *CrysAlis PRO* (Rigaku OD, 2018 ▸), *SHELXT2014/5* (Sheldrick, 2015*a* ▸), *SHELXL2017/1* (Sheldrick, 2015*b* ▸), *Mercury* (Macrae *et al.*, 2020 ▸) and *OLEX2* (Dolomanov *et al.*, 2009 ▸).

## supplementary crystallographic information

### Crystal data

**Table:**

C_36_H_37_ClN_4_O_7_·CH_4_O	*Z* = 2
*M**_r_* = 705.19	*F*(000) = 744
Triclinic, *P*1	*D*_x_ = 1.271 Mg m^−^^3^
*a* = 10.3211 (7) Å	Mo *K*α radiation, λ = 0.71073 Å
*b* = 13.9316 (11) Å	Cell parameters from 2243 reflections
*c* = 15.0269 (10) Å	θ = 3.6–24.8°
α = 107.179 (6)°	µ = 0.16 mm^−^^1^
β = 100.769 (6)°	*T* = 293 K
γ = 109.649 (7)°	Plate, colourless
*V* = 1843.2 (2) Å^3^	0.12 × 0.08 × 0.04 mm

### Data collection

**Table:**

Rigaku Xcalibur, Sapphire3 diffractometer	6484 independent reflections
Radiation source: fine-focus sealed X-ray tube	3993 reflections with *I* > 2σ(*I*)
Detector resolution: 16.1827 pixels mm^-1^	*R*_int_ = 0.062
ω scans	θ_max_ = 25.0°, θ_min_ = 3.0°
Absorption correction: multi-scan (CrysAlisPro; Rigaku OD, 2018)	*h* = −12→12
*T*_min_ = 0.718, *T*_max_ = 1.000	*k* = −16→16
12120 measured reflections	*l* = −17→17

### Refinement

**Table:**

Refinement on *F*^2^	14 restraints
Least-squares matrix: full	Hydrogen site location: mixed
*R*[*F*^2^ > 2σ(*F*^2^)] = 0.063	H atoms treated by a mixture of independent and constrained refinement
*wR*(*F*^2^) = 0.180	*w* = 1/[σ^2^(*F*_o_^2^) + (0.0804*P*)^2^] where *P* = (*F*_o_^2^ + 2*F*_c_^2^)/3
*S* = 0.98	(Δ/σ)_max_ < 0.001
6484 reflections	Δρ_max_ = 0.37 e Å^−^^3^
488 parameters	Δρ_min_ = −0.37 e Å^−^^3^

### Special details

**Table:**

Geometry. All esds (except the esd in the dihedral angle between two l.s. planes) are estimated using the full covariance matrix. The cell esds are taken into account individually in the estimation of esds in distances, angles and torsion angles; correlations between esds in cell parameters are only used when they are defined by crystal symmetry. An approximate (isotropic) treatment of cell esds is used for estimating esds involving l.s. planes.

### Fractional atomic coordinates and isotropic or equivalent isotropic displacement parameters (Å^2^)

**Table:**

	*x*	*y*	*z*	*U*_iso_*/*U*_eq_	Occ. (<1)
Cl1	0.18482 (10)	0.27201 (7)	0.45318 (8)	0.0879 (4)	
O1	0.1363 (2)	0.78835 (18)	0.78187 (15)	0.0656 (6)	
O2	0.6392 (2)	0.97400 (15)	0.79614 (13)	0.0527 (5)	
O3	0.7446 (2)	0.94517 (18)	0.63187 (18)	0.0595 (6)	
H3	0.741 (4)	0.957 (3)	0.586 (3)	0.083 (15)*	
O4	0.3890 (3)	0.87873 (18)	0.40675 (14)	0.0708 (7)	
O5	0.3359 (3)	0.30481 (19)	0.15424 (18)	0.0802 (8)	
O6	0.0559 (2)	0.67660 (18)	0.23246 (16)	0.0630 (6)	
O7	0.3038 (2)	0.61623 (18)	−0.17618 (14)	0.0609 (6)	
N1	0.1951 (3)	0.7675 (2)	0.70394 (18)	0.0612 (7)	
N2	0.4227 (2)	0.83021 (18)	0.68609 (15)	0.0460 (6)	
N3	0.3530 (2)	0.72925 (16)	0.27770 (14)	0.0392 (5)	
N4	0.0835 (3)	0.8240 (2)	0.19070 (17)	0.0490 (6)	
H4	0.152 (4)	0.884 (3)	0.195 (2)	0.074 (11)*	
C1	0.2010 (4)	0.8940 (3)	0.9544 (2)	0.0736 (10)	
H1A	0.128840	0.922066	0.943675	0.110*	
H1B	0.284465	0.949601	1.008583	0.110*	
H1C	0.161991	0.829562	0.968871	0.110*	
C2	0.2440 (3)	0.8644 (3)	0.8645 (2)	0.0546 (8)	
C3	0.3694 (3)	0.8946 (2)	0.8449 (2)	0.0531 (8)	
H3A	0.460502	0.945953	0.888417	0.064*	
C4	0.3337 (3)	0.8316 (2)	0.74402 (19)	0.0434 (7)	
C5	0.5651 (3)	0.9051 (2)	0.71337 (19)	0.0436 (7)	
C6	0.6065 (3)	0.8850 (2)	0.62320 (19)	0.0438 (7)	
C7	0.4959 (3)	0.8067 (2)	0.54664 (19)	0.0432 (7)	
C8	0.3665 (3)	0.7620 (2)	0.57990 (18)	0.0442 (7)	
H8	0.286697	0.776140	0.548349	0.053*	
C9	0.3169 (3)	0.6397 (2)	0.55535 (18)	0.0416 (7)	
C10	0.4071 (3)	0.5977 (2)	0.5935 (2)	0.0537 (8)	
H10	0.496737	0.646313	0.640469	0.064*	
C11	0.3678 (3)	0.4858 (2)	0.5639 (2)	0.0560 (8)	
H11	0.429826	0.458959	0.590555	0.067*	
C12	0.2347 (3)	0.4139 (2)	0.4940 (2)	0.0496 (7)	
C13	0.1416 (3)	0.4526 (2)	0.4563 (2)	0.0562 (8)	
H13	0.051140	0.403604	0.410486	0.067*	
C14	0.1827 (3)	0.5653 (2)	0.4868 (2)	0.0513 (7)	
H14	0.119336	0.591657	0.460966	0.062*	
C15	0.4877 (3)	0.7567 (2)	0.44215 (18)	0.0467 (7)	
H15A	0.441428	0.676778	0.419640	0.056*	
H15B	0.585368	0.777049	0.438053	0.056*	
C16	0.4042 (3)	0.7932 (2)	0.37513 (19)	0.0450 (7)	
C17	0.3430 (3)	0.6180 (2)	0.24142 (18)	0.0396 (6)	
C18	0.2315 (3)	0.5338 (2)	0.2456 (2)	0.0530 (8)	
H18	0.159408	0.548169	0.268223	0.064*	
C19	0.2238 (3)	0.4270 (2)	0.2165 (2)	0.0587 (8)	
H19	0.147233	0.370399	0.219647	0.070*	
C20	0.3309 (3)	0.4061 (2)	0.1831 (2)	0.0555 (8)	
C21	0.4416 (4)	0.4902 (3)	0.1765 (2)	0.0644 (9)	
H21	0.512658	0.475722	0.152511	0.077*	
C22	0.4478 (3)	0.5952 (2)	0.2053 (2)	0.0545 (8)	
H22	0.522867	0.651377	0.200380	0.065*	
C23	0.2286 (4)	0.2176 (3)	0.1641 (3)	0.0916 (13)	
H23A	0.135048	0.201790	0.122460	0.137*	
H23B	0.229750	0.238635	0.231160	0.137*	
H23C	0.247922	0.152986	0.145215	0.137*	
C24	0.2850 (3)	0.7726 (2)	0.21251 (18)	0.0404 (6)	
H24	0.342636	0.852694	0.238280	0.048*	
C25	0.2869 (3)	0.7253 (2)	0.10845 (19)	0.0421 (6)	
C26	0.4104 (3)	0.7724 (2)	0.0842 (2)	0.0512 (8)	
H26	0.491865	0.831257	0.132629	0.061*	
C27	0.4136 (3)	0.7330 (3)	−0.0107 (2)	0.0564 (8)	
H27	0.497132	0.764969	−0.025541	0.068*	
C28	0.2927 (3)	0.6460 (2)	−0.0838 (2)	0.0475 (7)	
C29	0.1706 (3)	0.5974 (2)	−0.0612 (2)	0.0533 (8)	
H29	0.089435	0.538224	−0.109724	0.064*	
C30	0.1692 (3)	0.6372 (2)	0.03492 (19)	0.0494 (7)	
H30	0.086484	0.603566	0.049952	0.059*	
C31	0.1776 (4)	0.5334 (3)	−0.2546 (2)	0.0748 (11)	
H31A	0.196560	0.524816	−0.315984	0.112*	
H31B	0.098515	0.554956	−0.254578	0.112*	
H31C	0.152642	0.464518	−0.246083	0.112*	
C32	0.1300 (3)	0.7536 (2)	0.21472 (19)	0.0434 (7)	
C33	−0.0649 (3)	0.8177 (3)	0.1762 (3)	0.0637 (9)	
C34	−0.1035 (4)	0.8284 (4)	0.2694 (3)	0.0987 (14)	
H34A	−0.104076	0.767117	0.286450	0.148*	
H34B	−0.197859	0.828863	0.259680	0.148*	
H34C	−0.033068	0.896180	0.321512	0.148*	
C35	−0.1715 (4)	0.7095 (3)	0.0922 (3)	0.1000 (14)	
H35A	−0.140232	0.702183	0.035187	0.150*	
H35B	−0.266155	0.709336	0.077334	0.150*	
H35C	−0.175204	0.648757	0.111037	0.150*	
C36	−0.0624 (5)	0.9159 (4)	0.1482 (4)	0.1060 (15)	
H36A	0.007832	0.983643	0.200456	0.159*	
H36B	−0.156610	0.916650	0.137701	0.159*	
H36C	−0.036727	0.908644	0.089156	0.159*	
O8A	0.754 (3)	1.031 (2)	0.4990 (12)	0.127 (8)	0.303 (10)
H8A	0.682895	1.041989	0.506038	0.191*	0.303 (10)
C37A	0.744 (3)	0.9975 (16)	0.3987 (13)	0.117 (6)	0.303 (10)
H37A	0.802378	1.059842	0.387193	0.175*	0.303 (10)
H37B	0.644924	0.969545	0.359214	0.175*	0.303 (10)
H37C	0.778623	0.940725	0.381619	0.175*	0.303 (10)
O8B	0.7271 (8)	0.9964 (6)	0.4729 (6)	0.084 (2)	0.697 (10)
H8B	0.655470	1.005555	0.450322	0.127*	0.697 (10)
C37B	0.8511 (8)	1.0727 (6)	0.4653 (7)	0.101 (3)	0.697 (10)
H37D	0.926332	1.046763	0.469376	0.151*	0.697 (10)
H37E	0.884329	1.143282	0.517811	0.151*	0.697 (10)
H37F	0.826852	1.079852	0.403396	0.151*	0.697 (10)

### Atomic displacement parameters (Å^2^)

**Table:**

	*U*^11^	*U*^22^	*U*^33^	*U*^12^	*U*^13^	*U*^23^
Cl1	0.0789 (6)	0.0378 (5)	0.1184 (8)	0.0190 (4)	0.0063 (6)	0.0136 (5)
O1	0.0552 (13)	0.0732 (16)	0.0524 (13)	0.0196 (12)	0.0169 (11)	0.0114 (11)
O2	0.0585 (12)	0.0386 (11)	0.0372 (11)	0.0093 (10)	0.0026 (10)	0.0033 (9)
O3	0.0551 (14)	0.0508 (14)	0.0535 (14)	0.0101 (11)	0.0117 (12)	0.0120 (11)
O4	0.1146 (19)	0.0506 (14)	0.0430 (12)	0.0494 (14)	0.0060 (12)	0.0071 (10)
O5	0.0854 (17)	0.0464 (15)	0.0958 (18)	0.0347 (13)	0.0134 (14)	0.0115 (13)
O6	0.0582 (13)	0.0572 (14)	0.0817 (16)	0.0205 (11)	0.0289 (12)	0.0376 (12)
O7	0.0605 (13)	0.0701 (15)	0.0466 (12)	0.0214 (11)	0.0230 (11)	0.0184 (10)
N1	0.0548 (16)	0.0588 (17)	0.0458 (14)	0.0111 (13)	0.0117 (13)	0.0052 (12)
N2	0.0468 (13)	0.0391 (14)	0.0361 (12)	0.0111 (11)	0.0070 (11)	0.0050 (10)
N3	0.0475 (13)	0.0298 (12)	0.0326 (11)	0.0155 (10)	0.0055 (10)	0.0070 (9)
N4	0.0488 (15)	0.0428 (15)	0.0511 (15)	0.0198 (13)	0.0066 (12)	0.0172 (12)
C1	0.073 (2)	0.087 (3)	0.054 (2)	0.032 (2)	0.0218 (18)	0.0199 (18)
C2	0.0558 (19)	0.053 (2)	0.0407 (16)	0.0199 (16)	0.0050 (15)	0.0088 (14)
C3	0.0506 (18)	0.0526 (19)	0.0384 (16)	0.0162 (15)	0.0043 (14)	0.0063 (13)
C4	0.0489 (17)	0.0353 (16)	0.0370 (14)	0.0144 (13)	0.0064 (13)	0.0099 (12)
C5	0.0529 (17)	0.0263 (14)	0.0381 (15)	0.0125 (13)	0.0017 (14)	0.0070 (12)
C6	0.0486 (17)	0.0333 (15)	0.0397 (15)	0.0123 (13)	0.0084 (13)	0.0102 (12)
C7	0.0485 (16)	0.0356 (15)	0.0371 (14)	0.0159 (13)	0.0032 (13)	0.0113 (12)
C8	0.0476 (16)	0.0343 (15)	0.0335 (14)	0.0125 (13)	−0.0001 (13)	0.0036 (11)
C9	0.0414 (15)	0.0370 (16)	0.0358 (14)	0.0112 (13)	0.0064 (12)	0.0093 (12)
C10	0.0450 (17)	0.0417 (18)	0.0537 (17)	0.0112 (14)	−0.0023 (14)	0.0102 (14)
C11	0.0502 (18)	0.047 (2)	0.065 (2)	0.0211 (15)	0.0082 (16)	0.0205 (15)
C12	0.0516 (17)	0.0326 (16)	0.0550 (17)	0.0135 (14)	0.0154 (15)	0.0097 (13)
C13	0.0469 (17)	0.0413 (18)	0.0533 (18)	0.0102 (14)	−0.0001 (14)	0.0015 (14)
C14	0.0445 (16)	0.0437 (18)	0.0499 (17)	0.0165 (14)	−0.0004 (14)	0.0089 (13)
C15	0.0612 (18)	0.0397 (16)	0.0347 (14)	0.0241 (14)	0.0075 (14)	0.0094 (12)
C16	0.0551 (17)	0.0311 (15)	0.0381 (15)	0.0162 (13)	0.0063 (13)	0.0065 (12)
C17	0.0427 (15)	0.0375 (16)	0.0327 (14)	0.0177 (13)	0.0058 (12)	0.0084 (11)
C18	0.0536 (18)	0.0432 (18)	0.0590 (19)	0.0194 (15)	0.0188 (15)	0.0159 (14)
C19	0.0585 (19)	0.0346 (18)	0.068 (2)	0.0119 (15)	0.0101 (17)	0.0143 (15)
C20	0.062 (2)	0.0386 (18)	0.0523 (18)	0.0274 (16)	0.0026 (16)	0.0020 (14)
C21	0.067 (2)	0.056 (2)	0.075 (2)	0.0359 (18)	0.0266 (19)	0.0170 (17)
C22	0.0540 (18)	0.0471 (19)	0.0589 (19)	0.0210 (15)	0.0161 (16)	0.0174 (14)
C23	0.097 (3)	0.043 (2)	0.113 (3)	0.028 (2)	0.004 (3)	0.020 (2)
C24	0.0437 (15)	0.0331 (15)	0.0366 (14)	0.0128 (12)	0.0044 (12)	0.0121 (11)
C25	0.0468 (16)	0.0378 (16)	0.0384 (14)	0.0150 (13)	0.0099 (13)	0.0154 (12)
C26	0.0442 (17)	0.0541 (19)	0.0468 (17)	0.0149 (14)	0.0071 (14)	0.0189 (14)
C27	0.0449 (17)	0.068 (2)	0.0549 (19)	0.0178 (16)	0.0179 (15)	0.0269 (16)
C28	0.0540 (18)	0.0513 (18)	0.0412 (16)	0.0246 (15)	0.0173 (14)	0.0188 (14)
C29	0.0538 (18)	0.0444 (18)	0.0424 (16)	0.0069 (14)	0.0121 (14)	0.0089 (13)
C30	0.0496 (17)	0.0475 (18)	0.0383 (15)	0.0087 (14)	0.0160 (14)	0.0116 (13)
C31	0.087 (3)	0.071 (2)	0.0449 (18)	0.013 (2)	0.0268 (19)	0.0129 (17)
C32	0.0462 (16)	0.0418 (17)	0.0360 (14)	0.0166 (14)	0.0091 (13)	0.0111 (12)
C33	0.0533 (19)	0.059 (2)	0.071 (2)	0.0274 (17)	0.0067 (17)	0.0194 (17)
C34	0.074 (3)	0.119 (4)	0.106 (3)	0.051 (3)	0.038 (2)	0.030 (3)
C35	0.066 (2)	0.088 (3)	0.099 (3)	0.023 (2)	−0.017 (2)	0.011 (2)
C36	0.092 (3)	0.101 (3)	0.153 (4)	0.061 (3)	0.020 (3)	0.074 (3)
O8A	0.140 (12)	0.122 (14)	0.101 (11)	0.012 (11)	0.040 (9)	0.065 (10)
C37A	0.178 (15)	0.106 (12)	0.098 (11)	0.062 (11)	0.086 (10)	0.050 (9)
O8B	0.088 (4)	0.073 (4)	0.096 (6)	0.033 (3)	0.032 (3)	0.036 (3)
C37B	0.107 (6)	0.111 (6)	0.137 (7)	0.060 (5)	0.068 (5)	0.081 (5)

### Geometric parameters (Å, º)

**Table:**

Cl1—C12	1.741 (3)	C18—H18	0.9300
O1—N1	1.417 (3)	C18—C19	1.391 (4)
O1—C2	1.348 (3)	C19—H19	0.9300
O2—C5	1.224 (3)	C19—C20	1.377 (4)
O3—H3	0.75 (4)	C20—C21	1.377 (5)
O3—C6	1.346 (3)	C21—H21	0.9300
O4—C16	1.221 (3)	C21—C22	1.373 (4)
O5—C20	1.371 (3)	C22—H22	0.9300
O5—C23	1.409 (4)	C23—H23A	0.9600
O6—C32	1.225 (3)	C23—H23B	0.9600
O7—C28	1.365 (3)	C23—H23C	0.9600
O7—C31	1.425 (4)	C24—H24	0.9800
N1—C4	1.307 (3)	C24—C25	1.514 (4)
N2—C4	1.380 (3)	C24—C32	1.540 (4)
N2—C5	1.381 (3)	C25—C26	1.393 (4)
N2—C8	1.474 (3)	C25—C30	1.377 (4)
N3—C16	1.357 (3)	C26—H26	0.9300
N3—C17	1.443 (3)	C26—C27	1.378 (4)
N3—C24	1.482 (3)	C27—H27	0.9300
N4—H4	0.87 (3)	C27—C28	1.384 (4)
N4—C32	1.335 (4)	C28—C29	1.373 (4)
N4—C33	1.475 (4)	C29—H29	0.9300
C1—H1A	0.9600	C29—C30	1.390 (4)
C1—H1B	0.9600	C30—H30	0.9300
C1—H1C	0.9600	C31—H31A	0.9600
C1—C2	1.486 (4)	C31—H31B	0.9600
C2—C3	1.337 (4)	C31—H31C	0.9600
C3—H3A	0.9300	C33—C34	1.505 (5)
C3—C4	1.412 (4)	C33—C35	1.522 (5)
C5—C6	1.473 (4)	C33—C36	1.539 (5)
C6—C7	1.322 (4)	C34—H34A	0.9600
C7—C8	1.515 (4)	C34—H34B	0.9600
C7—C15	1.491 (4)	C34—H34C	0.9600
C8—H8	0.9800	C35—H35A	0.9600
C8—C9	1.507 (4)	C35—H35B	0.9600
C9—C10	1.379 (4)	C35—H35C	0.9600
C9—C14	1.388 (4)	C36—H36A	0.9600
C10—H10	0.9300	C36—H36B	0.9600
C10—C11	1.375 (4)	C36—H36C	0.9600
C11—H11	0.9300	O8A—H8A	0.8200
C11—C12	1.381 (4)	O8A—C37A	1.412 (5)
C12—C13	1.364 (4)	C37A—H37A	0.9600
C13—H13	0.9300	C37A—H37B	0.9600
C13—C14	1.381 (4)	C37A—H37C	0.9600
C14—H14	0.9300	O8B—H8B	0.8200
C15—H15A	0.9700	O8B—C37B	1.412 (5)
C15—H15B	0.9700	C37B—H37D	0.9600
C15—C16	1.513 (4)	C37B—H37E	0.9600
C17—C18	1.366 (4)	C37B—H37F	0.9600
C17—C22	1.383 (4)		
			
C2—O1—N1	108.9 (2)	C20—C21—H21	119.7
C6—O3—H3	106 (3)	C22—C21—C20	120.5 (3)
C20—O5—C23	117.9 (3)	C22—C21—H21	119.7
C28—O7—C31	117.5 (2)	C17—C22—H22	119.8
C4—N1—O1	104.6 (2)	C21—C22—C17	120.4 (3)
C4—N2—C5	125.3 (2)	C21—C22—H22	119.8
C4—N2—C8	122.4 (2)	O5—C23—H23A	109.5
C5—N2—C8	111.2 (2)	O5—C23—H23B	109.5
C16—N3—C17	121.8 (2)	O5—C23—H23C	109.5
C16—N3—C24	115.9 (2)	H23A—C23—H23B	109.5
C17—N3—C24	121.48 (19)	H23A—C23—H23C	109.5
C32—N4—H4	114 (2)	H23B—C23—H23C	109.5
C32—N4—C33	125.7 (3)	N3—C24—H24	107.3
C33—N4—H4	119 (2)	N3—C24—C25	112.2 (2)
H1A—C1—H1B	109.5	N3—C24—C32	111.1 (2)
H1A—C1—H1C	109.5	C25—C24—H24	107.3
H1B—C1—H1C	109.5	C25—C24—C32	111.3 (2)
C2—C1—H1A	109.5	C32—C24—H24	107.3
C2—C1—H1B	109.5	C26—C25—C24	119.8 (2)
C2—C1—H1C	109.5	C30—C25—C24	122.5 (2)
O1—C2—C1	115.8 (3)	C30—C25—C26	117.8 (3)
C3—C2—O1	109.4 (3)	C25—C26—H26	119.5
C3—C2—C1	134.7 (3)	C27—C26—C25	121.0 (3)
C2—C3—H3A	127.4	C27—C26—H26	119.5
C2—C3—C4	105.1 (3)	C26—C27—H27	119.9
C4—C3—H3A	127.4	C26—C27—C28	120.2 (3)
N1—C4—N2	118.8 (2)	C28—C27—H27	119.9
N1—C4—C3	111.9 (3)	O7—C28—C27	115.9 (3)
N2—C4—C3	129.3 (3)	O7—C28—C29	124.3 (3)
O2—C5—N2	126.0 (3)	C29—C28—C27	119.8 (3)
O2—C5—C6	128.1 (3)	C28—C29—H29	120.3
N2—C5—C6	106.0 (2)	C28—C29—C30	119.5 (3)
O3—C6—C5	117.3 (2)	C30—C29—H29	120.3
C7—C6—O3	131.8 (3)	C25—C30—C29	121.8 (3)
C7—C6—C5	110.9 (2)	C25—C30—H30	119.1
C6—C7—C8	109.5 (2)	C29—C30—H30	119.1
C6—C7—C15	130.0 (3)	O7—C31—H31A	109.5
C15—C7—C8	120.3 (2)	O7—C31—H31B	109.5
N2—C8—C7	102.3 (2)	O7—C31—H31C	109.5
N2—C8—H8	109.6	H31A—C31—H31B	109.5
N2—C8—C9	114.2 (2)	H31A—C31—H31C	109.5
C7—C8—H8	109.6	H31B—C31—H31C	109.5
C9—C8—C7	111.2 (2)	O6—C32—N4	124.3 (3)
C9—C8—H8	109.6	O6—C32—C24	121.5 (3)
C10—C9—C8	121.5 (2)	N4—C32—C24	114.0 (3)
C10—C9—C14	117.9 (3)	N4—C33—C34	110.8 (3)
C14—C9—C8	120.4 (2)	N4—C33—C35	109.5 (3)
C9—C10—H10	119.2	N4—C33—C36	105.1 (3)
C11—C10—C9	121.7 (3)	C34—C33—C35	111.2 (3)
C11—C10—H10	119.2	C34—C33—C36	110.1 (3)
C10—C11—H11	120.5	C35—C33—C36	109.9 (3)
C10—C11—C12	119.0 (3)	C33—C34—H34A	109.5
C12—C11—H11	120.5	C33—C34—H34B	109.5
C11—C12—Cl1	119.7 (2)	C33—C34—H34C	109.5
C13—C12—Cl1	119.4 (2)	H34A—C34—H34B	109.5
C13—C12—C11	120.9 (3)	H34A—C34—H34C	109.5
C12—C13—H13	120.3	H34B—C34—H34C	109.5
C12—C13—C14	119.4 (3)	C33—C35—H35A	109.5
C14—C13—H13	120.3	C33—C35—H35B	109.5
C9—C14—H14	119.4	C33—C35—H35C	109.5
C13—C14—C9	121.1 (3)	H35A—C35—H35B	109.5
C13—C14—H14	119.4	H35A—C35—H35C	109.5
C7—C15—H15A	109.0	H35B—C35—H35C	109.5
C7—C15—H15B	109.0	C33—C36—H36A	109.5
C7—C15—C16	112.7 (2)	C33—C36—H36B	109.5
H15A—C15—H15B	107.8	C33—C36—H36C	109.5
C16—C15—H15A	109.0	H36A—C36—H36B	109.5
C16—C15—H15B	109.0	H36A—C36—H36C	109.5
O4—C16—N3	121.5 (2)	H36B—C36—H36C	109.5
O4—C16—C15	121.5 (2)	C37A—O8A—H8A	109.5
N3—C16—C15	116.9 (2)	O8A—C37A—H37A	109.5
C18—C17—N3	120.0 (2)	O8A—C37A—H37B	109.5
C18—C17—C22	118.9 (3)	O8A—C37A—H37C	109.5
C22—C17—N3	121.0 (3)	H37A—C37A—H37B	109.5
C17—C18—H18	119.4	H37A—C37A—H37C	109.5
C17—C18—C19	121.3 (3)	H37B—C37A—H37C	109.5
C19—C18—H18	119.4	C37B—O8B—H8B	109.5
C18—C19—H19	120.4	O8B—C37B—H37D	109.5
C20—C19—C18	119.2 (3)	O8B—C37B—H37E	109.5
C20—C19—H19	120.4	O8B—C37B—H37F	109.5
O5—C20—C19	124.3 (3)	H37D—C37B—H37E	109.5
O5—C20—C21	116.0 (3)	H37D—C37B—H37F	109.5
C19—C20—C21	119.7 (3)	H37E—C37B—H37F	109.5
			
Cl1—C12—C13—C14	−178.3 (2)	C9—C10—C11—C12	0.2 (5)
O1—N1—C4—N2	179.9 (2)	C10—C9—C14—C13	−1.2 (4)
O1—N1—C4—C3	−0.3 (3)	C10—C11—C12—Cl1	178.4 (2)
O1—C2—C3—C4	−0.4 (3)	C10—C11—C12—C13	−1.6 (5)
O2—C5—C6—O3	3.6 (4)	C11—C12—C13—C14	1.7 (5)
O2—C5—C6—C7	−177.3 (3)	C12—C13—C14—C9	−0.2 (5)
O3—C6—C7—C8	176.7 (3)	C14—C9—C10—C11	1.2 (4)
O3—C6—C7—C15	1.0 (5)	C15—C7—C8—N2	177.6 (2)
O5—C20—C21—C22	−178.9 (3)	C15—C7—C8—C9	55.3 (3)
O7—C28—C29—C30	−177.4 (3)	C16—N3—C17—C18	78.2 (3)
N1—O1—C2—C1	−178.7 (3)	C16—N3—C17—C22	−99.5 (3)
N1—O1—C2—C3	0.2 (3)	C16—N3—C24—C25	156.7 (2)
N2—C5—C6—O3	−176.9 (2)	C16—N3—C24—C32	−78.1 (3)
N2—C5—C6—C7	2.2 (3)	C17—N3—C16—O4	−166.9 (3)
N2—C8—C9—C10	−54.2 (4)	C17—N3—C16—C15	16.2 (4)
N2—C8—C9—C14	131.3 (3)	C17—N3—C24—C25	−33.4 (3)
N3—C17—C18—C19	−176.2 (2)	C17—N3—C24—C32	91.9 (3)
N3—C17—C22—C21	176.0 (3)	C17—C18—C19—C20	0.1 (5)
N3—C24—C25—C26	−83.4 (3)	C18—C17—C22—C21	−1.7 (4)
N3—C24—C25—C30	97.7 (3)	C18—C19—C20—O5	178.8 (3)
N3—C24—C32—O6	−27.8 (3)	C18—C19—C20—C21	−1.6 (5)
N3—C24—C32—N4	155.6 (2)	C19—C20—C21—C22	1.5 (5)
C1—C2—C3—C4	178.2 (4)	C20—C21—C22—C17	0.2 (5)
C2—O1—N1—C4	0.1 (3)	C22—C17—C18—C19	1.6 (4)
C2—C3—C4—N1	0.5 (4)	C23—O5—C20—C19	−3.3 (5)
C2—C3—C4—N2	−179.8 (3)	C23—O5—C20—C21	177.1 (3)
C4—N2—C5—O2	10.4 (5)	C24—N3—C16—O4	3.0 (4)
C4—N2—C5—C6	−169.1 (2)	C24—N3—C16—C15	−173.9 (2)
C4—N2—C8—C7	168.3 (2)	C24—N3—C17—C18	−91.1 (3)
C4—N2—C8—C9	−71.4 (3)	C24—N3—C17—C22	91.1 (3)
C5—N2—C4—N1	168.8 (3)	C24—C25—C26—C27	−178.1 (3)
C5—N2—C4—C3	−10.9 (5)	C24—C25—C30—C29	177.5 (2)
C5—N2—C8—C7	−0.1 (3)	C25—C24—C32—O6	97.9 (3)
C5—N2—C8—C9	120.2 (3)	C25—C24—C32—N4	−78.7 (3)
C5—C6—C7—C8	−2.2 (3)	C25—C26—C27—C28	0.5 (5)
C5—C6—C7—C15	−177.9 (3)	C26—C25—C30—C29	−1.5 (4)
C6—C7—C8—N2	1.4 (3)	C26—C27—C28—O7	177.0 (3)
C6—C7—C8—C9	−120.9 (3)	C26—C27—C28—C29	−1.4 (5)
C6—C7—C15—C16	−107.2 (3)	C27—C28—C29—C30	0.9 (4)
C7—C8—C9—C10	60.9 (3)	C28—C29—C30—C25	0.6 (5)
C7—C8—C9—C14	−113.6 (3)	C30—C25—C26—C27	0.9 (4)
C7—C15—C16—O4	22.5 (4)	C31—O7—C28—C27	−174.8 (3)
C7—C15—C16—N3	−160.6 (2)	C31—O7—C28—C29	3.6 (4)
C8—N2—C4—N1	2.2 (4)	C32—N4—C33—C34	61.9 (4)
C8—N2—C4—C3	−177.5 (3)	C32—N4—C33—C35	−61.1 (4)
C8—N2—C5—O2	178.3 (3)	C32—N4—C33—C36	−179.1 (3)
C8—N2—C5—C6	−1.2 (3)	C32—C24—C25—C26	151.4 (2)
C8—C7—C15—C16	77.5 (3)	C32—C24—C25—C30	−27.5 (4)
C8—C9—C10—C11	−173.5 (3)	C33—N4—C32—O6	−3.6 (4)
C8—C9—C14—C13	173.5 (3)	C33—N4—C32—C24	172.9 (2)

### Hydrogen-bond geometry (Å, º)

**Table:**

*D*—H···*A*	*D*—H	H···*A*	*D*···*A*	*D*—H···*A*
O3—H3···O8*A*	0.75 (4)	1.88 (5)	2.61 (3)	162 (4)
O3—H3···O8*B*	0.75 (4)	1.93 (4)	2.680 (10)	175 (4)
N4—H4···O2^i^	0.87 (3)	2.33 (3)	3.193 (3)	170 (3)
O8*A*—H8*A*···O4^i^	0.82	1.88	2.60 (3)	145
O8*B*—H8*B*···O4^i^	0.82	2.50	2.843 (8)	107

Symmetry code: (i) −*x*+1, −*y*+2, −*z*+1.
